# Forensic Interpretation and Importance of Simon’s Bleeding, Amussat’s Sign and Other Typical Findings of Hanging as Diagnostic Signs

**DOI:** 10.7759/cureus.57809

**Published:** 2024-04-08

**Authors:** Biliana Mileva, Metodi Goshev, Martina Valcheva, Alexandar Alexandrov, Ilina Braynova

**Affiliations:** 1 Department of Forensic Medicine and Deontology, Medical University Sofia, Sofia, BGR

**Keywords:** asphyxia, strangulation, vital signs, diagnostic challenges, suicide, forensic pathology, hanging, amussat's sign, simon's sign

## Abstract

Hanging is amongst the most commonly used methods of suicide. Multiple findings could be observed in such cases. Knowing the proper mechanism of their occurrence and how to interpret them is of utmost importance in forensic practice to avoid misinterpretation and wrong conclusions. The study aimed to present a case of hanging with putrefactive changes - external and internal findings, signs or prolonged stay in a hanged position, their interpretation, and discussion as part of the routine forensic practice.

## Introduction

Hanging refers to a compression of the neck by means of ligature constricted by the weight of the body. It is amongst the most commonly used methods of suicide since a vast number of objects and materials in everyday life could be used as ligature material. Secondly, following the compression of the neck, the hanged person loses consciousness in a short period of time and does not feel pain [1]. Hanging can be divided into two main categories depending on the body's position, whether it is freely hanged or not. When the body is freely hanged, there is no contact of any of its body parts with the ground or other nearly situated surfaces, and this is known as complete hanging. Opposite to this, when the body has some form of contact with the ground or the surroundings, it is known as incomplete hanging or partial suspension [1,2]. A different mechanism could provoke death, either by compression of the carotid arteries and jugular veins leading to cerebral ischemia, compression of the airways leading to asphyxia, or by stimulation of the carotid sinuses, usually, they are superimposed [3,4]. In rare occasions, injury of the cervical spine with spinal cord disruption may occur, leading to complete or incomplete decapitation as a result of suicidal hanging [1,5-7]. The last is usually a consequence of the so-called long drop (about 4-5 m). In a short drop (0,5-1 m), there is a lower prevalence of cervical spine injuries [2,8].

## Case presentation

A woman in her 40s was found freely hanged in the corridor of her apartment. The ligature material was a rope tightened around the neck, with a knot on the left side. There was information that she was suffering from depression, and she was isolating herself from the others. Her brother was trying to call her consistently for a few days prior to discovering her dead body, and when she didn’t answer her phone, he decided to visit her apartment and found her hanged. The apartment was locked from the inside, and nothing out of the ordinary was found; it was not messy, and there was no evidence of a struggle or attempted theft. The body was sent for an autopsy, which was performed the following day.

**Figure 1:**
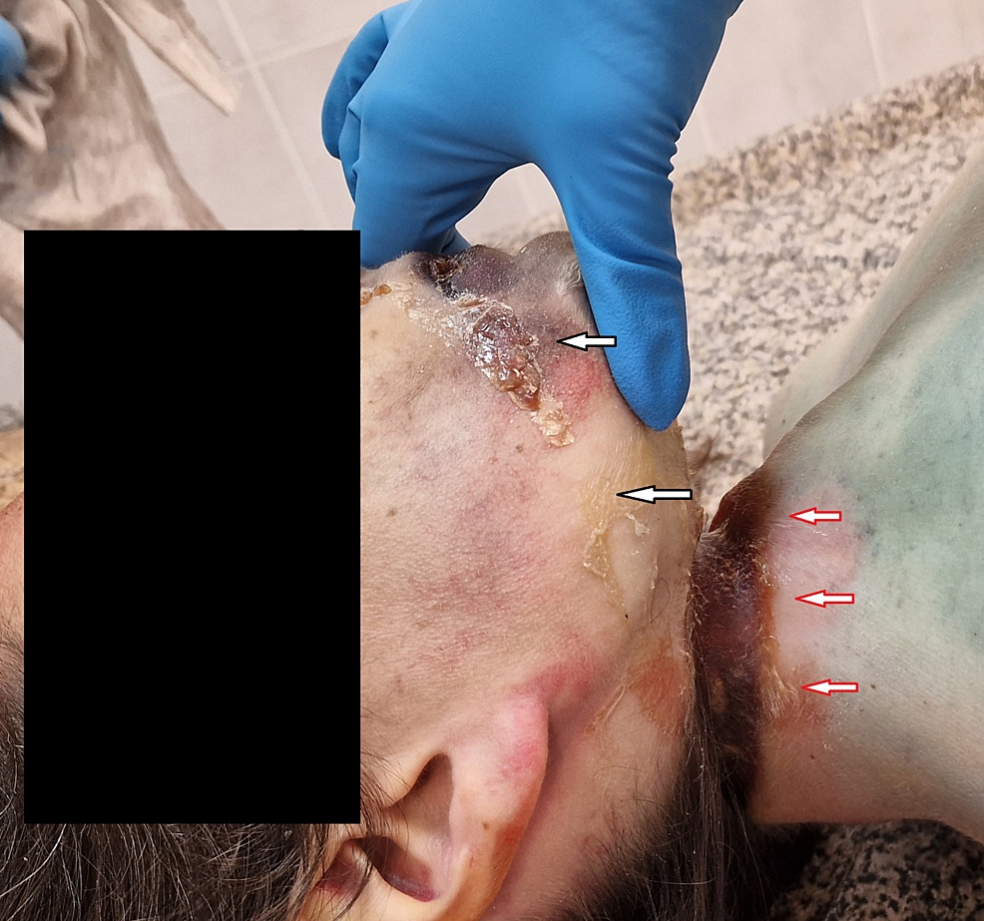
External examination image Ligature mark (red arrows); Dribbling of saliva (white arrows).

The corpse was normally dressed and the clothes were without suspicious staining or tears. At the external examination, the lividities were intensive, with typical localization in the abdominal area, encircling all the surfaces of the lower limbs and the distal parts of the upper extremities, and they were fixed. In the same area, visible signs of decomposition were present, comprising greenish discoloration of the skin, with some areas of skin slippage and marbling. The upper part of the body was bright, without lividities, and with bright greenish discoloration at some places. The lips were desiccated with dark brownish discoloration. Dribbling of saliva was present, dried up in the form of a bright yellowish crust from the right angle of the mouth, opposite side of the knot. No traumatic injuries were found over the deceased body, apart from the ligature mark encircling the neck. The last was more pronounced on the right side of the neck, and from that side, the formed furrow was tracked obliquely upward and disappeared behind the left ear. The neck in the zone of the ligature mark was significantly thinned, and its circumference was measured around 20-21 cm. The sustained ligature mark was leathery, dark brown to yellowish, with a pattern corresponding to the one of the rope (Figure 1).

**Figure 2:**
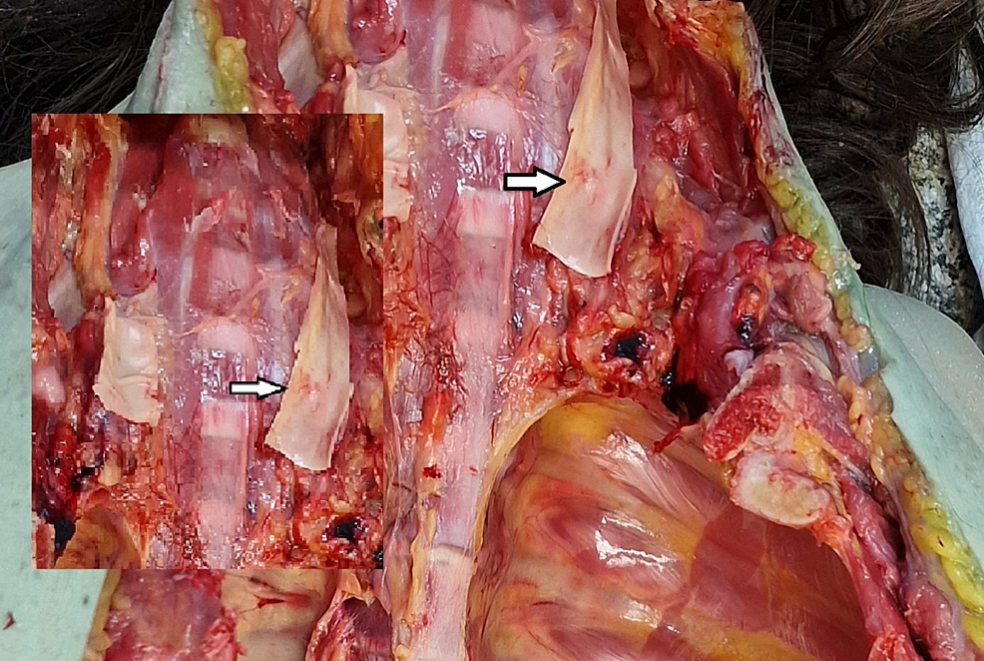
Amussat's sign Minimal transversal intimal tear of the left common carotid artery - Amussat's sign.

**Figure 3:**
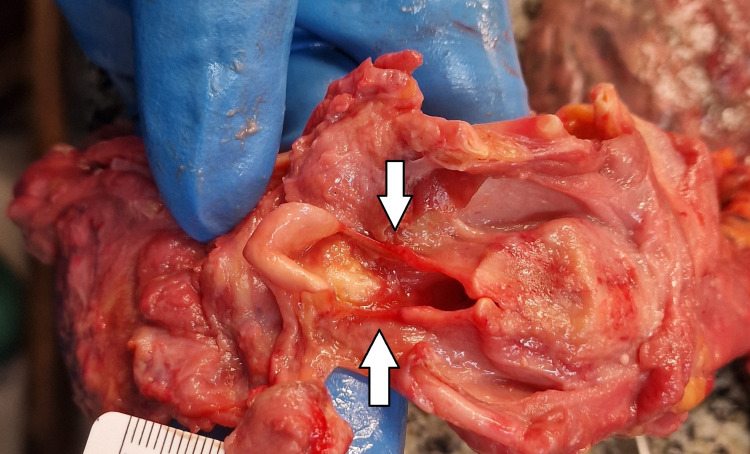
Overstretching of the epiglottis

**Figure 4:**
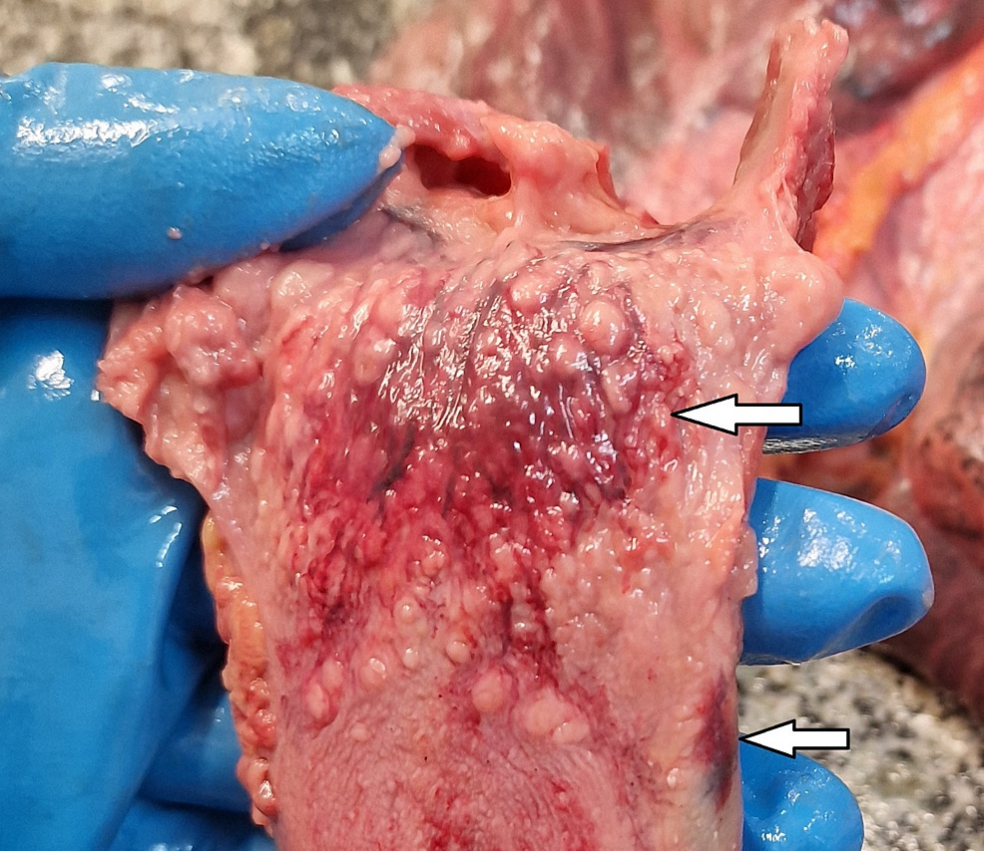
Signs on the tongue Hemotthages at the base of the tongue and bruise on the left side of the tongue.

**Figure 5:**
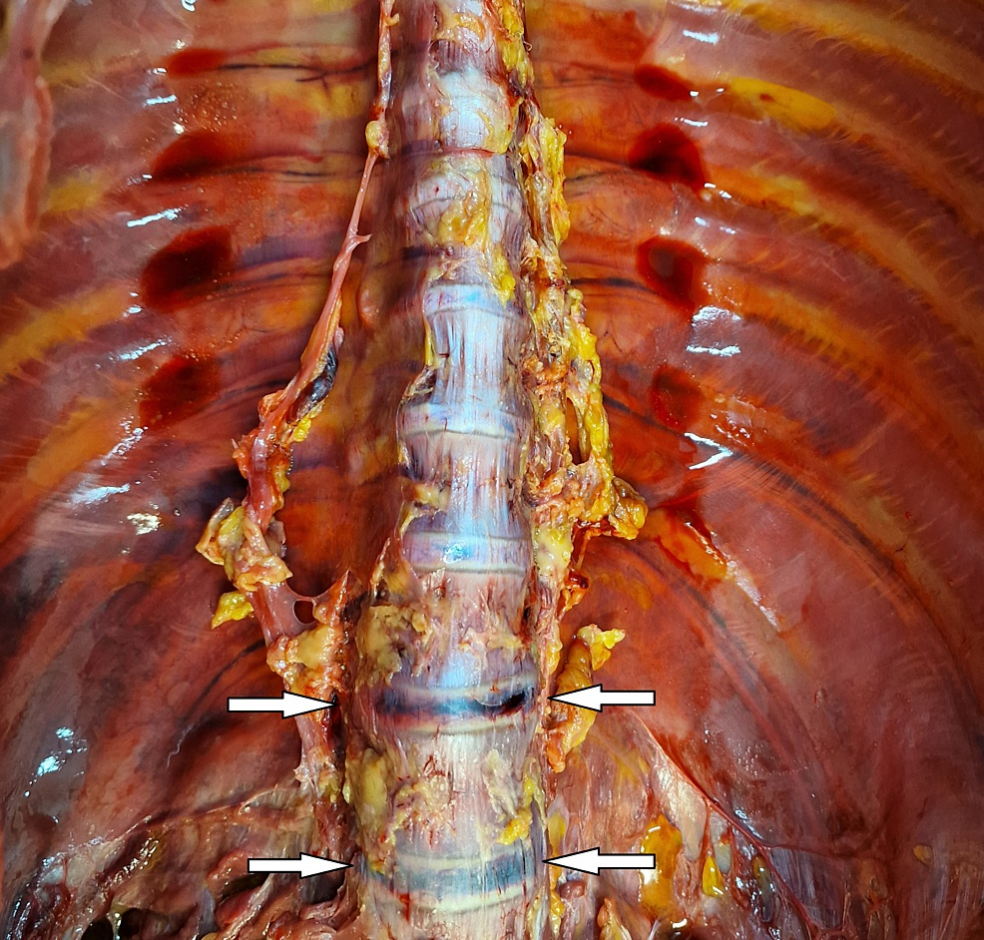
Simon's bleeding Bleeding on the anterior aspects of the intervertebral discs of the lumbar region-Simon's bleeding.

The internal examination of the body revealed the following abnormal findings: Minimal transversal intimal tear of the left common carotid artery, known as Amussat’s sign (Figure 2); the epiglottis looked overstretched with a dried appearance in the zone of “elongation” (Figure 3); hemorrhages at the base of the tongue, as well as small bruise at the left side of the tongue (Figure 4); fracture of the hyoid bone, bleeding on the anterior aspects of the intervertebral discs of the lumbar region, consistent with Simon’s bleeding (Figure 5), dot-like hemorrhages under the pleura of the lungs.

No other findings were noted during the autopsy that were associated with trauma or disease. The internal organs had preserved a gross appearance, congested, with minor autolytic changes. Blood samples and urine were taken for toxicological analyses, and the results were negative. The cause of death was attributed to mechanical asphyxia from compression of the neck-hanging. The manner of death was declared to be suicide.

## Discussion

The main task of the forensic pathologist in cases of hanging is to determine whether the hanging occurred while the person was alive or if the body of the deceased was placed intentionally to simulate hanging to hide homicide [9]. Depending on the level of compression, and the force applied, there are few typical findings in cases of hanging, which are proven to be vital signs in cases of hanging [9]. Depending on the level of compression, fractures of the hyoid bone and thyroid cartilage with bruises of the surrounding soft tissues might occur [1,10]. Hemorrhages in the sternocleidomastoid muscles' origins may be seen on one or both sides and are mostly more acutely pronounced on the side on which the ligature’s highest point is located [1]. Another typical finding in hanging cases is unilateral or bilateral transversal intimal tears of the carotid artery [1,4,6]. The most probable mechanism for Amussat’s sign is a combination of direct compression of the artery by the ligature material and indirect due to overstretching of the vessel produced by the weight of the body [1,4]. The finding does not represent a vital sign [1]. In some cases, authors report fresh hemorrhages in pharyngeal tonsils and the tongue, which could be caused by the base of the tongue pressing against the roof of the pharynx due to compression of the floor of the mouth caused by the weight of the body on the tie used for hanging [9,11].

A relatively common finding in hanging is the so-called “Simon’s bleedings”, “Simon’s sign”, or “Simon’s hemorrhages”. These are fine red or purple streaks of hemorrhages observed beneath the anterior longitudinal ligament in intervertebral discs [1,3,4]. They are limited only to the area of the anterior ligament of intervertebral disks and do not penetrate into the vertebral bodies [3,4]. The German forensic pathologist Axel Simon was the first to describe them in 1968 [3,4,10]. The most common localization of the bleeding is in the lumbosacral region of the spine since this is the most flexible region [2]. The last results from the rupture of the capillary bed of either the lumbar arteries or veins [4]. The mechanism of occurrence of this finding is a combination of two factors, the overstretching by the weight of the body in combination with the terminal agonal convulsions typical for death from asphyxia [1,3,4,10,12]. The last was proved by the study of Nikolić and Zivković which showed that Simon’s bleeding was present in many other types of mechanical asphyxiation [8]. Although Simon’s bleeding is mostly associated with complete hanging, there are described cases where it is present in case of incomplete hanging [12]. Additionally, there are reported cases in the literature of Simon’s sign associated with blunt force trauma, where the last results from the direct effect of the blunt force over the specific region of the spine with hyperextension or hyperflexion of the spinal column [13,14]. Authors have found a significant difference between the occurrence of Simon’s bleeding in people of different ages. Those findings are rare in individuals with severe degenerative changes in the lumbosacral region of the spinal column since the last makes it less flexible and protects the blood vessels from rupture [3,4,10]. Simon’s hemorrhages are considered signs of vitality [1,3-5,10]. Although reported and described in different cases and regarded as a vital reaction in situations where decomposition is established, those findings might be simulated due to hemolysis [1].

In the case presented, the cause of death was attributed to hanging, which was confirmed by the presence of the typical findings: (a) The presence of a ligature mark, which corresponds to the specific patterns of the ligature used, (b) The dribbling of saliva, (c) The bruises on the base of the tongue, (d) The fracture of the hyoid bone, (e) The transversal intimal tear of the left carotid artery, (f) The presence of Simon’s sign, and (g) The congestion of the internal organs with petechial hemorrhages under the pleura of the lungs.

A mark from saliva is reported as a vital reaction and is explained as a consequence of the stimulation and irritation of submandibular salivary glands that only occurs during life, due to pressing and friction from the ligature [9,15]. The congestion of the internal organs with petechial hemorrhages under the conjunctivas of the eyes or the serous membranes of the internal organs is associated with the petechiae/congestion syndrome, which is often observed in a different type of asphyxia but is not specific and is seen in various cases of sudden death as well [1]. There were no other traumatic injuries, apart from the strangulation mark, over the deceased body, which could explain the cause of death or eventually be suspected of defense wounds in a case of struggle.

The findings that are supportive for a prolonged period of hanging are the fixed lividities with typical localization of the lower parts of the body and more advanced decomposition of these regions of the body due to the settling down of the blood in the depending areas of the body due to the gravity; the small circumference of the neck and the overstretching of the epiglottis with its drying. The authors believe that a possible postmortem decapitation was likely if the body was discovered later and that these could be marked as its early signs or “pre-decapitation signs”.

## Conclusions

Concluding about the cause of death in asphyxia-related deaths, especially in a case where decomposition is present, has to be performed with special attention, and following a thorough and detailed analysis of all the information concerning the case-death scene investigation, medical history, autopsy findings, and toxicology. Although many of the "typical" findings of hanging and asphyxia, in general, could be observed in many other types of deaths, their presence in combination gives valuable information and supports the forensic pathologist's conclusion about the cause of death.
